# Analytical Strategies Involved in the Detailed Componential Characterization of Biooil Produced from Lignocellulosic Biomass

**DOI:** 10.1155/2017/9298523

**Published:** 2017-12-13

**Authors:** Yao Lu, Guo-Sheng Li, Yong-Chao Lu, Xing Fan, Xian-Yong Wei

**Affiliations:** ^1^Key Laboratory of Coal Processing and Efficient Utilization, Ministry of Education, China University of Mining & Technology, Xuzhou 221116, China; ^2^Advanced Analysis & Computation Center, China University of Mining & Technology, Xuzhou 221116, China; ^3^School of Chemical and Engineering Technology, China University of Mining & Technology, Xuzhou 221116, China; ^4^School of Basic Education Sciences, Xuzhou Medical University, Xuzhou 221004, China

## Abstract

Elucidation of chemical composition of biooil is essentially important to evaluate the process of lignocellulosic biomass (LCBM) conversion and its upgrading and suggest proper value-added utilization like producing fuel and feedstock for fine chemicals. Although the main components of LCBM are cellulose, hemicelluloses, and lignin, the chemicals derived from LCBM differ significantly due to the various feedstock and methods used for the decomposition. Biooil, produced from pyrolysis of LCBM, contains hundreds of organic chemicals with various classes. This review covers the methodologies used for the componential analysis of biooil, including pretreatments and instrumental analysis techniques. The use of chromatographic and spectrometric methods was highlighted, covering the conventional techniques such as gas chromatography, high performance liquid chromatography, Fourier transform infrared spectroscopy, nuclear magnetic resonance, and mass spectrometry. The combination of preseparation methods and instrumental technologies is a robust pathway for the detailed componential characterization of biooil. The organic species in biooils can be classified into alkanes, alkenes, alkynes, benzene-ring containing hydrocarbons, ethers, alcohols, phenols, aldehydes, ketones, esters, carboxylic acids, and other heteroatomic organic compounds. The recent development of high resolution mass spectrometry and multidimensional hyphenated chromatographic and spectrometric techniques has considerably elucidated the composition of biooils.

## 1. Introduction

Fossil resources, namely, coal, petroleum, and natural gas, are still the main raw materials to meet the global requirements for energy and fine chemicals. The fast depletion and increasing demands increase price of fossil resources, leading to serious economic and social crises, which strongly motivates the search for alternative and renewable resources [1]. Lignocellulosic biomass (LCBM), which is considered as the most abundant renewable and low-cost organic resource with global production of 15 M tons per year, is the most promising choice [2]. LCBM can be degraded via thermochemical and biological techniques to produce solid, liquid, and gaseous fuels [3–5], which provides about 15% of the world's primary energy consumption and is treated as an additional CO_2_ neutral process. On the other hand, from the chemical point of view, the main parts of LCBM are cellulose, hemicellulose, and lignin [3, 6]. Cellulose is a linear polysaccharide of*β*-(1→4)-D-glucopyranose with degree of polymerization around 5000–10000. Hemicellulose is a heterogeneous polysaccharide mixture of various polymerized monosaccharides (i.e., glucose, mannose, galactose, xylose, arabinose, etc.) with a degree of polymerization around tens to hundreds. Lignin is an amorphous, three-dimensional, highly branched polyphenolic substance consisting of an irregular array of hydroxy-/methoxy-substituted phenylpropane units supported by *β*-O-4, *α*-O-4, 5-5, *β*-5, and *β*-*β* linkages [3, 7, 8].

Currently, no process can be accepted exclusively and considered as the superior option for the thermochemical conversion of LCBM into liquid fuels and organic chemicals. Pyrolysis is a promising conversion method and attracts increasing attention in the last three decades because it is more suitable for the direct production of a second-generation liquid fuel [3, 9–13]. Pyrolysis of dry LCBM is a thermal degradation process in the absence of O_2_, at high temperature (i.e., >350°C), with short residence times (several seconds) and under specific pressure (especially N_2_, >1 MPa), which produces major content of biooil and biochar, as well as small amount of gas. Biooil can be used as fuel and feedstock of value-added chemicals directly or with simple pretreatments [3]. To maximize the biooil yield from various LCBMs, the optimization of pyrolysis parameters such as the residence time, heating rate, temperature, and pressure is applied [3, 10, 14, 15]. In order to use biooil in value-added ways, more detailed information regarding the composition of biooil should be carried out [16–19]. The compositions of the degradation products from different LCBMs are extremely complex [3, 20, 21] and depend on the kind, part, or growth stage of LCBM [20]. A significant factor affecting the composition of biooils is the pyrolytic conditions such as pretreatment method, reactor style, heating rate, quenching rate, residence time, and temperature [3, 10, 11, 14, 15, 22]. Different from biodiesel and bioethanol, biooils are produced from pyrolysis of LCBM, resulting in dark brown oily liquid with extremely complex compositions, including water and hundreds of organic species which can be classified into hydrocarbons (alkanes, alkenes, alkynes, and aromatics), oxygen-containing species (ethers, alcohols, phenols, aldehydes, ketones, furans, esters, and carboxylic acids), and other heteroatomic organic compounds [21]. Classes of organic components contained in biooils are listed in Table 1.

The compositional analysis of biooil covers a wide range of its characteristics, including macroscopical and integral features such as solid-liquid phases distribution, acidity, stability, heating value, elemental contents, and molecular weight distribution and microscopical and specific features such as chemical composition, distribution of moieties of species, distributions of functional groups and chemical bonds, and connection styles of organic matters. Most of these analyses require qualitative analysis and quantitative analysis. The physicochemical fuel properties of biooil, such as water content, elemental contents, heating value, acidity, and density, as well as phase distribution, are determined by corresponding techniques which are similar with the conventional methods used in the evaluation of petroleum-based fuels [16, 23, 24]. Typical values for the physicochemical fuel properties of biooil are shown in Table 2. The water content of biooil is often determined by Karl Fischer titration method. The solid content is determined from the residue after filtering the biooil through a polyethersulfone syringe filter. The contents of C, H, O, N, and P in biooil are often analyzed through complete oxidation with an elementary analyzer. Gel permeation chromatography (GPC) is often used to measure the size and molecular weight of macromolecules. The methods used in the physicochemical fuel properties determination of biooil are not included in this review.

Some of the undesirable properties [33] for the utilization as fuel may include high water content, high viscosity, high corrosiveness (high acidity), high oxygen content, low heating value, and multiphase instability (polymerization reaction attributed to the presence of aldehydes and phenols). Thus, the biooil upgrading with economical sustainable techniques aiming at improving the physicochemical properties as fuel is necessary. From the viewpoint of production of value-added chemicals, upgrading processes leading to economic profit are also needed to be carried out [34–37]. In situ heterogeneous catalysis during pyrolysis and separated catalytic hydrotreatment (hydrodeoxygenation) are promising methods used in the upgrading of biooil production [38–41]. After these processes, yield of conversion, ratio of light weight moieties, and stability can be increased, and acidity and oxygen content can be decreased. The upgrading methods are not included in this review, because there is no obvious difference on componential characterization methods between the biooil and upgraded biooil [18, 42, 43].

Insight into the detailed compositional characterization of LCBM derived biooil is crucial for the development of efficient conversion processes and better upgrading strategies, for the evaluation of value-added utilization of biooil, for the understanding of the composition of LCBM, and for the probing of the degradation mechanisms in pyrolytic process. The final goal of compositional characterization is to produce fuels and chemicals to meet the demand for energy and chemical feedstock. The elucidation of composition of biooil or upgraded biooil brings great analytical challenge. Due to the complexity and diversity of the components, it is impossible to completely characterize a biooil by a single analytical method [44]. Typically, biooils contain more than 300 organic compounds which could be detected by gas chromatography-mass spectrometry (GC-MS) [3, 21]. This number of organic species only accounts for a small part of the full components due to the determination limit of GC-MS, which only works for species with lower boiling point (<350°C) and low to medium polarity [45, 46]. A report stated that more than 8000 peaks were characterized in a biooil with a high resolution mass spectrometer (HRMS) [47].

The chemical compositions of biooils are extremely complex; hence, specific, comprehensive, and robust analytical methodologies should be used. Several review works have been carried out for the detailed compositional characterization of biooil [17, 18, 48, 49]. To determine the functional groups and chemical bonds distribution, and individual species in biooil, distinct separation and detection methodologies were applied. After preseparation of moieties in biooil [21, 33, 49], such as liquid-liquid extraction (LLE), distillation, and column chromatography, both spectroscopic and chromatographic methods are applied to the qualitative analysis and quantitative analysis of the species in biooil [17, 44, 50–53]. For spectroscopic methods, Fourier transform infrared (FTIR) spectroscopy [29, 54–56] and nuclear magnetic resonance (NMR) spectroscopy [56–59] are mostly used, which can provide information on the functional groups of species in biooil. However, such information is integral information, and not for a specific compound. Thus, the separation and isolation of individual components are needed. Based on physicochemical properties of the components that need to be analyzed, that is, solubility, volatility, molecular weight, and ionization potential, chromatographic techniques can provide effective componential separation and give the qualitative and quantitative estimation for individual species. Some of the majority of components in biooils have very low concentrations (*i.e.*, <0.2 wt%), and a detailed compositional analysis requires the combination of separation techniques. HPLC [60–65] and GC [21, 33, 66–69] allow primary qualitative and quantitative classification of the detectable components and are the most commonly available techniques used for effective separation of species for complex samples. GC usually concentrates on the volatile organic species with lower polarity and lower boiling point (<350°C) with molecular weight range 50–500 Da typically, and HPLC is usually used for the separation of species with lower vapor pressures, lower thermal stability, and higher polarity. However, only a portion of the sample is identified. The other components with higher molecular weight (MW) can be determined, that is, by GPC for species with MW up to 1000–2000 Da without any further information regarding their structure [25, 60], unless coupled with other techniques [70]. Comprehensive two-dimensional gas chromatography (GC × GC) is a powerful technique for the determination of volatile fractions in different types of biooils providing detailed information on the molecular composition. It provides complementary qualitative and quantitative analyses of a wide variety of compounds [25, 71–76]. Both the chromatographic methods are linked to various detectors, such as atomic emission detector (AED) [77], flame ionization detector (FID) [73, 78], and various mass spectrometers [25, 71–74, 79, 80]. A single technique cannot provide complete analysis. Different detectors have different sensitivities and resolutions to specific species and elements, also with inherent limitations. For example, AED is less sensitive to oxygen than other heteroatoms [77, 81], and MS is more sensitive to species easy to be ionized [19, 69, 72–74, 82]. GC × GC with higher resolution over GC has been successfully used for the separation of components in various biooils produced from different LCBMs. However, the identification of individual species is a difficult task since comprehensive databases and retention time libraries are not widely available. Improved analytical methods were emerged to take advantage on the characterization of species in biooil. HRMSs were extensively used to identify individual component according to the monoisotopic mass in complex mixtures [17, 44, 47, 74, 76, 83, 84].

Sometimes, the chromatographic and spectrometric methods are not sufficient to identify all the species with similar retention behavior and spectra since their limited resolution. To overcome this problem and for better qualitative and quantitative analyses of species, internal standard and external standard methods are often applied by using reference compounds [21, 78, 85, 86]. However, not all the compounds are synthetically or commercially available due to the complexity, namely, hundreds of species contained in the pyrolytic biooils. Therefore, more powerful techniques aiming at exact identification of organic species in complex samples with outstanding resolution are needed. HRMSs, such as Orbitrap MS, Q-TOFMS, and Fourier transform-ion cyclotron resonance mass spectrometry (FT-ICR MS), were reported to have advantage on the identification of complex mixtures due to the superior mass resolution [17, 27, 28, 30, 31, 44, 47, 70, 74, 76, 83, 87, 88].

In the current review, analytical strategies involved in the detailed componential characterization of biooil produced from LCBM in recent years were reviewed. Pretreatments of biooil before further compositional analyses were summarized, and the instrumental analyses for detailed characterization of biooil samples with chromatographic and spectrometric methods especially the hyphenated chromatographic techniques, comprehensive NMR spectroscopy, and HRMS techniques were highlighted. These compositional assessments were compared and discussed, and the future work on the componential characterization of biooil was also prospected and suggested.

## 2. Production of Pyrolytic Biooils

Pyrolysis is a process of thermal decomposition of dry LCBM in absence of O_2_, leading to the cleavage of chemical bonds for the production of biooil, biochar, and gas, with controlled conditions,* that is*, programmed temperature, residence time, and pressure and with/without a catalyst [3]. The distribution of these products changing with pyrolysis parameters, and the maximum liquid fraction yield can be obtained under optimized conditions. Slow, fast, and flash pyrolysis are the three main methods used in the degradation of LCBM according to the pyrolytic conditions. The main product of slow pyrolysis is biochar with typical pyrolytic conditions,* that is*, temperature 350–500°C, heating rate about 10°C/s, and residence time 5–30 min. Typical conditions for fast pyrolysis are as follows,* that is*, temperature 500–650°C, heating rate about 100°C/s, and residence time 0.5–5 s. The formation of biooil, biochar, and gas with different yields varies with starting material and conditions used in the pyrolysis. Under the vigorous pyrolytic conditions,* that is*, temperature 700–1000°C, heating rate up to 10000°C/,s and residence time < 0.1 s, flash pyrolysis takes place with the main product of gas. Among the three pyrolysis methods, fast pyrolysis is a promising technique for the conversion of biomass to fuels because of relatively mild conditions, effective degradable ability, and appropriate distribution of products. One of the disadvantages of fast pyrolysis is that considerable amount of lignin in LCBM could not be decomposed effectively, which is left in the biochar as residue [3].

Some pyrolysis processes with modification or optimization are similar with in situ upgrading of biooil aiming at improving the fuel properties and/or utilization as feedstock of chemicals [12, 22]. The type of pyrolysis reaction system such as microwave-assisted pyrolysis and stepwise pyrolysis system also influences the yield and composition of biooil [89–92]. Catalytic pyrolysis of LCBM with various catalysts, including activated carbon [93–95], and alkaline catalysts [96, 97], for the production of biooil, has been carried out [98].

Typically, biooils contain 15–30 wt% of water, 55–65 wt% of organic components that can be quantified with conventional chromatographic and spectrometric techniques [3], and 5–30 wt% components with condensed structure and high MW cannot be detected by conventional methods [17, 44].

## 3. Fractionation Techniques

For the complexity of biooils, sample pretreatments focused on fractionation were often employed to achieve more detailed information or to impart selectivity and specificity in componential analysis. Pretreatments are typically applied to biooils to obtain fractions according to the difference in properties of components before the subsequent chromatographic analysis. Among the methods used in pretreatment, extraction, distillation, filtration, centrifugation, and adsorption chromatography have been widely explored for various applications [17, 44, 49, 98, 99].  Table 3 lists the regular techniques used in the pretreatment before componential analysis of biooil.

### 3.1. Solvent Extraction

Solvent extraction, also known as LLE, is a process in which solvent is used for recovery of components from biooil. In a typical process, the addition of a relatively immiscible organic solvent to the sample results in two phases, namely, extract phase and raffinate phase, also known as solvent-rich phase and water-rich phase, respectively. Based on the difference of solubility or affinity between the two phases, redistribution of the components takes place according to their distribution or partition coefficient. Commonly used solvents for the extraction include water, methanol, ethanol, ethyl acetate, acetonitrile,* n*-hexane, benzene and toluene, ketones, dichloromethane, and carbon tetrachloride [21, 28, 29, 33, 99, 100]. By using hexane, petroleum ether, or chloroform as the extraction solution in LLE, phenols, and guaiacol were enriched into the solvent phase with high concentrations (85%), while sugar, acid, and alcohol were concentrated into the water phase [100]. For complex mixtures with multiple moieties of components to be extracted, such as biooil, selected solvents extraction can be performed for multiple stages [101]. Hence, in typical solvent extraction procedure, in order to conduct the extraction of moieties of components effectively, large volumes of solvents and tedious extraction program are required, making it a time consuming process [17].

Supercritical fluid extraction (SFE) offers several advantages compared to conventional solvent extraction and has received much attention applied for the componential separation of biooil [102–105]. CO_2_, the solvent in SFE, is inexpensive, nontoxic, nonflammable, noncorrosive, and readily available in large quantities with high purity. The SFE process with CO_2_ is generally carried out at relatively low temperature and low pressure, because the low critical pressure (*i.e.*, 73.8 atm) and critical temperature (*i.e.*, 31.1°C) of CO_2_ prevent undesirable reactions among biooil components. Continuous modulation of the conditions in the process of SFE is flexible for selective extraction of the supercritical fluid; furthermore, it is easy to remove CO_2_ from the extracts after SFE, preventing undesirable pollution during the extraction. The organic fraction of a biooil produced from mixed biomass of wheat and wood sawdust was isolated by supercritical CO_2_ (SC-CO_2_), and the first fraction of SC-CO_2_ extraction collected at 25 MPa was enriched with furanoids (9.9%), pyranoids (9.0%), and benzenoids (44.8%) [102]. Cheng et al. [106] carried out a three-step SC-CO_2_ extraction for the selective fractionation of fast pyrolysis biooil. Different classes of oxygen-containing compounds in biooil were enriched in different fractions, facilitating the following detailed analysis.

### 3.2. Distillation

Distillation usually follows LLE for further treatment of extracts, such as purification of products and recovery of solvents [21, 28, 29, 33]. In distillation of biooil, separation is based on the volatilities of components such as the vapor pressures and boiling points of the species. According to the characteristics of components and the purpose of distillation, atmospheric pressure, vacuum, steam, and molecular distillation can be applied flexibly to the separation. Under atmospheric pressure, due to the composition of biooil, with temperature lower than 100°C as the starting point, the distillation of biooil would be stopped at 280°C and left 35–50 wt% of the original material as a residue [107]. Distilled fractions with high purity can be obtained by using vacuum distillation (VD). VD can be conducted at much lower temperatures, and the vapor pressures of components decrease significantly according to the vacuum degree used in the process. By introducing steam into the distilling column to heat biooil and decrease its viscosity, the steam distillation allows thermally sensitive components to be separated avoiding conversion. Molecular distillation (MD) has some advantages compared to conventional distillations, such as lower operating temperatures, shorter heating time, and higher separation efficiency [108–110]. The principle of MD is based on the specific mean free paths of molecules, rather than difference in their boiling points. Different fractions of biooil could be enriched with various chemical families by MD. Small molecules formed a water-rich light fraction and monophenols accumulated in the middle fraction, while sugars and phenolic oligomers remained in the heavy fraction because of their high MWs [108–110].

Hydrotreated biooils with different oxygen contents can be distilled to produce lights (<71°C), naphtha (71–182°C), jet (182–260°C), diesel (260–338°C), and gas oil (338–566°C) boiling range fractions to enhance the detailed characterization of oxygen-containing species with advanced analytical instruments [63]. Carboxylic acids and carbonyl compounds were detected in fractions with boiling point under 260°C,* that is*, in the lights, naphtha, and jet fractions. A new approach to the fractionation of biooil by temperature-swing extraction was reported by Kumar et al. [111], in which hot extraction (around 70°C) of the light fraction with a suitable extraction solvent followed by cold (around 25°C) demixing of the light fraction and the extraction solvent allows solvent regeneration by spontaneous liquid/liquid phase split upon cooling. They illustrated a broader potential of utilization in the fractionation of crude oil [111].

### 3.3. Column Chromatography

Column chromatography is a conventional method to fractionate biooils according to the different adsorption capabilities of biooil components onto the stationary phase, prior to chromatographic or spectrometric analysis. Silica gel and aluminum oxide are often used as stationary phase. Organic solvents, such as pentane, benzene, carbon disulfide, toluene, dichloromethane, ethyl acetate, and methanol, are often used as elutes to isolate species in biooils into aliphatic, aromatic, and polar fractions and so forth [14, 69, 112–115]. However, the main disadvantages of column chromatography are low throughput making it only suitable for high value-added compounds [116], and the consuming of large amount of solvent as elute.

### 3.4. Other Chromatographic Techniques

**Figure 1:**
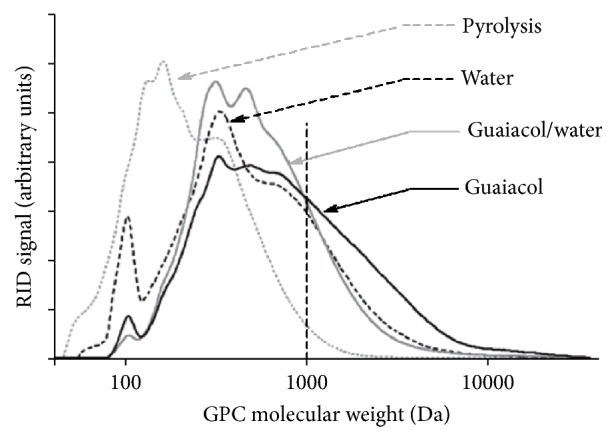
GPC results of the isolated biooils with guaiacol, guaiacol/water, and water, respectively [25].

The principles of chromatographic techniques are based on difference of components in the interaction with stationary phase in the chromatography column, inducing the difference of retention time. GPC was frequently used in the fractionation of species and the MW distribution analysis of biooil [15, 25, 117, 118]. A detailed GPC analysis of MW distribution in biooil was carried out for different fractions [25], as shown in Figure 1. Other chromatographic techniques, such as adsorption chromatography (AC) [60, 119], ion-exchange chromatography (IEC) [120, 121], and size exclusion chromatography (SEC) [122, 123], are potentially used in the preseparation or fractionation of components in biooil. AC, like thin layer chromatography [60, 119] with providing retention behavior of species, based on the weak interactions between molecules and stationary phase, such as van der Waals forces and steric attraction. Solvents, such as* n*-pentane, toluene, and methanol, were often used to isolate aliphatic, aromatic, and polar fractions, readily for further characterization. IEC is based on the adsorption of charged molecules or ions on the surface of ion-exchange resins due to the attraction induced by electrostatic forces. High polar species, such as hydrolysable sugars in water or acidic solutions, can be qualified with IEC [120, 121]. In SEC, packing material with a certain size of pores is used as stationary phase, and the separation of components is based on the difference in penetration of molecules according to their size and shape [122, 123].

## 4. Advanced Instrumental Strategies

The most commonly used technique to identify biooil components is GC-MS. However, GC-MS is limited to qualifying short-chain and/or nonpolar compounds, depending on the chromatographic column used in GC, and derivatization is usually required to analyze polar species [33]. Thermostable components with low MW can be analyzed by GC-MS owing to the low injector temperature in GC. HPLC is often used to identify polar and high MW species providing complementary information with GC for the more detailed elucidation of components in biooil [58, 60, 61]. Detectors coupled with GC and HPLC cover a wide range,* that is*, variable wavelength detector (VWD), diode array detector (DAD), FID, electron capture detection (ECD), nitrogen phosphorus detector (NPD), fluorescence detector (FLD), flame photometric detector (FPD), MS, and so forth. Among them, MS has exhibited flexible application in the organic compounds detection by varying ionization sources and mass analyzers. HRMSs are treated as ideal techniques for componential analysis of complex mixtures due to considerable resolving power [17, 27, 28, 30, 31, 44, 47, 74, 76, 87]. Comprehensive chromatographic techniques such as GC × GC [75–77, 124] and LC × LC [65] with high throughput for identification of components in complex mixtures attract more and more attention recently. Spectrometric techniques, such as FTIR and NMR [58, 74, 125] with various types (*i.e.*, ^1^H, ^13^C, and ^31^P), providing both qualitative and quantitative results were applied in the characterization of functional groups in biooils. Moreover, two-dimensional NMR (2D NMR) [59] is another promising technique used in the detailed characterization of components in biooil.

### 4.1. FTIR

FTIR is the most conventional technique used in the qualitative and quantitative analyses of organic substance for the determination of functional groups in almost all areas of contemporary chemical and biological researches [21, 29, 33, 54, 55, 69, 115]. In practice, mid-infrared region is extensively utilized to reveal the presence of various structures in molecules because of significant characteristic absorptions of functional groups. FTIR analysis is simple, straightforward, and useful for fast componential evaluation of biooil. Biooil contains enormous species, from hydrocarbons and alcohols to aromatics and carboxylic acids, so the spectra of biooil may include most of the characteristic peaks [126, 127]. Generally, peaks around 3050 cm^−1^ with strong absorbance are attributed to C-H stretching vibration indicating the presence of aliphatic hydrocarbons. Peaks around 3300–3400 cm^−1^ correspond to O-H stretching vibrations indicating the presence of carboxylic acids and/or alcohols. Peaks between 1450 and 1600 cm^−1^ indicate a C=C stretching vibration caused by aliphatic or aromatic structure. A significant peak between 1600 and 1800 cm^−1^ attributes to the presence of C=O containing species, such as aldehydes, ketones, carboxylic acids, and/or esters. Peak between 1000 and 1100 cm^−1^ is assigned to C-O stretching indicating the presence of ethers, alcohols, carboxylic acids, and/or esters. Since the table containing characteristic stretching wavelengths corresponding to functional groups can be easily found in textbooks, handbooks, and other publications [17], it is not concluded in this review.

Thermogravimetric analysis (TGA) coupled to FTIR, known as TGA-FTIR, was often used in the analysis of pyrolytic biooil [128–131]. TGA-FTIR shows the accurate weight loss of feedstock with time and provides the information on functional groups of the volatile species produced during the pyrolysis [132]. The three-dimensional TGA-FTIR spectra including infrared absorbance, wave number, and temperature showed that many volatile compounds are released in the pyrolysis of dried rice husk. Spectral intensity as a function of time can be obtained when the wave number is fixed. This information can be used to analyze the generation of specific components [129]. Stankovikj and Garcia-Perez [131] proposed a new method to identify the position and shape of the peaks of chemical families in the derivative thermogravimetry (DTG) curves and further to estimate the changes in the content of water, light volatile compounds, and water-soluble/insoluble fractions.

Due to the advantages of FTIR analysis, it can be used in biooil analysis for understanding molecular characteristics, evaluation of upgrading process, judging the further utilization, distinguishing biooils from different LCBMs, and so forth.

### 4.2. GC and Comprehensive Gas Chromatography (GC × GC)

GC-FID quantification of hydrocarbons can be performed with a high degree of accuracy using the effective carbon number (ECN) approach [60, 133–135], even when no authentic standard is available. Relative response factors (RRFs) could be calculated by correlation with the chemical formula and were used to qualify compounds in biooil with GC-FID [53, 136]. When considering MW, molecular formula, chemical composition, functional group, and chromatographic retention time, better results could be obtained [60].

Although GC-MS is extensively employed for the componential analysis of biooils, one of the challenges encountered in GC-MS analysis is assay of the mass spectrum of species. The resulting chromatograms are complicated due to the overlapping of peaks and the identification of components relied heavily on the basis of the NIST database. Furthermore, similarities among spectra of substituted species increase the difficulty of identification. Nevertheless, only part of the species could be characterized in the sample. In order to enhance the separation, pretreatments were carried out to concentrate or enrich moieties of species of interest by extraction, distillation, column chromatography, and so forth.

To obtain high peak capacity and resolution, and to gain accurate quantification of the individual components, GC × GC is often used in the compositional analysis of complex samples. GC × GC techniques have the advantage of increased chromatographic resolving power, allowing the detection and characterization of many classes of compounds [71–77, 85–87, 124, 137–139]. The separation is carried out on two columns with different polarities, and a device called modulator located between the two columns transfers the effluent periodically. For a GC × GC system, orthogonal system consists of a nonpolar column and a polar one, while a nonorthogonal system has the opposite combination, called reversed phase system. After simple pretreatments, such as dilution, extraction, and adsorption, biooils can be conducted in a GC × GC system with various detectors including ECD, FID, and MSs. Based on the analysis of GC × GC, retention behavior of species to be determined is plotted along the* x*-/*y*-axis with respect to the two columns concluded in the dispersion graphics (DGs), and the varying color or contour lines of spots represent the intensity of the peaks. Identification and classification of species are performed via statistic and chemometric methods. The qualitative and quantitative approaches of grouped classes of species are demonstrated by the peak density and intensity in different areas on the DGs.

The quantification of species in biooil is based on internal [140] or external standard calibration method [86] with chosen reference compounds, depending on the detector used and the targeted compounds. External calibration can provide accurate quantitative analysis for selected species. However, the analysis is limited to only a few species in the samples because only a small part of species in biooil are commercially available and the concentrations of interested species vary in a wide range. Internal calibration method is a much more versatile quantification method in which an internal standard is chosen as a reference compound with similar structure, retention behavior, and ionization efficiency, compared to the analyte. Based on the RRF of each compound and the internal standard, internal standard quantification can be applied to a wide range of classes of species for quantitative analysis, whatever the variation of sample preparation and analytical instrument [60].

**Figure 2:**
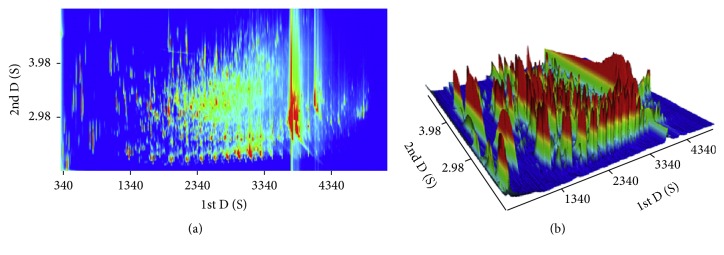
Typical GC × GC/TOFMS analysis of crude pyrolysis oil. (a) Topographic map and (b) tridimensional view [26].

The use of GC × GC, frequently coupled with TOFMS [74] or Q-MS [124], is a promising combination used in the qualitative analysis of biooils for the detailed characterization of compounds. Relatively, GC × GC/TOFMS provides a higher number of identified species compared to GC/Q-MS [141]. Typical 3D plot for componential analysis of biooils using GC × GC/TOFMS is presented in Figure 2. This is most likely attributed to the coelution of species resulting in the overlapping of peaks in GC-MS. Using GC × GC/TOFMS, semiquantitative analysis [26, 142] of samples can be performed using a relative area of detected compounds with a signal-to-noise ratio higher than 1000 and the total of identified compounds in each sample was higher than 80%. With similar method, biooils from catalytic pyrolysis of pine wood and sugarcane bagasse were analyzed, and the results showed that acids, ketones/cyclic ketones, phenols, and O-heterocyclic and aromatic hydrocarbons were the main components [74]. Zhang et al. [124] carried out chemical characterization of crude and ethanol-stabilized biooils before and after accelerated aging treatment using a GC × GC/TOFMS to discuss stabilization mechanism of the addition of ethanol into biooil. Twenty-six standards (C_7_–C_32_* n*-alkanes) in the calibration mixture were used to test instrument capability and evaluate selected quality control parameters. There were 2728, 2212, 2674, and 2781 peaks identified in the crude biooil before/after aging and the ethanol-stabilized biooil before/after aging, respectively, and the major component groups were ketones, phenols, furanones, and acids. The addition of ethanol and accelerated aging treatment could both slightly change the chemical composition of biooils. By using GC × GC/TOFMS, more detailed information could be obtained for comprehensive understanding of internal mechanism of ethanol addition and aging treatment on the storage stability of pyrolysis biooil. Purcaro et al. [73] constructed a GC × GC system coupled with simultaneous dual detectors, FID and MS, for quantitative and qualitative analyses of minor compounds in vegetable oils. Such technique proved to be effective not only in a qualitative viewpoint but also for quantitative purposes, especially for investigation of minor compounds in a single run.

The identification of species was significantly improved by coupling several sensitive detectors or using advanced data handling methods. Meanwhile, the combined analytical system with higher resolution needs to be set up, and the quantitative analysis methods also need to be established.

### 4.3. HPLC and Two-Dimensional Liquid Chromatography (LC × LC)

Compared to GC, HPLC is good at characterizing nonvolatile, unstable, and high MW compounds in the fractions of biooil products. However, the resolution may be poorer than GC due to the length limitation of column and other separation conditions used. Coupled with MS, HPLC is also the extensively used combination for analysis of components in biooil with high sensitivity for species easy to be ionized.

Various HPLC detectors have been used for analyte characterization. Carbonyl compounds, such as aldehydes and ketones, can be qualified by HPLC-UV after derivation with 2,4-dinitrophenylhydrazine (DNPH) [63, 143]. By HPLC, coupled with evaporative light scattering detector (ELSD) [144], triglyceride molecular species of the extracted oil were identified by their ECN and the elution order was tentatively predicted according to fatty acid composition [145]. As mentioned above, MS detectors including HRMS (e.g., TOFMS, Orbitrap, and FT-ICR) were extensively used in the characterization of components in biooil. More than 400 compounds with MWs mainly distributed between 100 and 400 Da were identified in a biooil by HPLC-Orbitrap MS [60].

The LC × LC separates samples comprehensively via elution in two columns connected in series [65, 146–148]. The establishment of two dimensions is based on either stationary phase in the two columns with different separation mechanisms or elute. Similar with GC × GC system, a modulator or switch (also called interface) was mounted between the two columns. The modulator transfers elute from the primary column to the secondary column quickly with high pressure inside the separation system. The fraction injected into the secondary column should be completely analyzed before the successive transfer occurs, while the second dimension analysis time should be at least equal to or less than the duration of a modulation period. However, because of the retention space being delimited by the retention times of the least and the most retained compounds in both dimensions [146–148], the separation of biooil extracts by LC × LC with a percentage of retention space covers around 50%. Detectors used in the conventional LC system, such as UVD, PDA, ELSD, and MS, can be applied in LC × LC system by direct on-line coupling. Tomasini et al. [148] demonstrated the efficiency of LC×LC equipped with PDA for the componential analysis of a dihydroxygenated biooil with a non-silica-based column and a sub-2 *μ*m silica-based column for the primary and the second dimension, respectively. They prospected number of detectable species up to 2000.

### 4.4. Mass Spectrometry

MS are often used as detector coupled with chromatographic techniques for the assignment of components in biooils [31, 47, 149–151]. Ionization methods of MS include electrospray ionization (ESI), atmospheric pressure photoionization (APPI), atmospheric pressure chemical ionization (APCI), electron ionization (EI), chemical ionization (CI), fast atom bombardment (FAB), laser desorption ionization (LDI), and matrix assisted laser desorption ionization (MALDI). The former three methods were commonly used due to their mild conditions at either positive or negative mode, also called “soft” ionization methods. ESI is more effective for the detection of polar compounds with lower MWs, while APCI and APPI are more applicable to identify nonpolar molecules [152–154]. Among the ionization methods reported, ESI with negative mode has been used mostly to detect a relative wider range of compounds in biooils. Recent study showed that the combination of ionization methods was more suitable for the characterization of species in biooils [59, 155]. With ESI/APPI FT-ICR MS, furfural derivatives, phenolics, aliphatics, and oligomers were well detected in hydrothermal liquefaction production of glucose and cellulose as model compounds [59]. After comparison of two ionization methods, namely, ESI and APCI, a combination of ESI and APCI at their negative modes were found to be well suited for the characterization of biooils. In the studied biooils, mostly compounds with 1–8 oxygen atoms per molecule were detected and their degree of unsaturation (double-bond equivalence, DBE) was about 1–10 (ESI) and 1–17 (APCI), respectively [155].

In recent years, in order to enhance the sensitivity and selectivity, direct-infusion mass spectrometry (DIMS) analysis has been widely used to detect and identify many chemical compounds in different matrices [156–158], such as liquids produced of pyrolysis and hydrothermal liquefaction. Some advantages of this technique are minimal sample preparation steps, faster analysis, and a wider range of compounds detected at the same injection.

HRMS analysis is based on the accurate mass measurement with sufficiently high mass resolving power for the compounds with different elemental composition. The mass resolving power of HRMS refers to the ability of increasing the mass accuracy with low* m*/*z* measurement error allowing separating two narrow mass spectral peaks with similar MW. Generally, the required mass resolving power and accuracy of the mass measurement depend on the complexity of the analyzed sample and the purpose of analysis.

**Figure 3:**
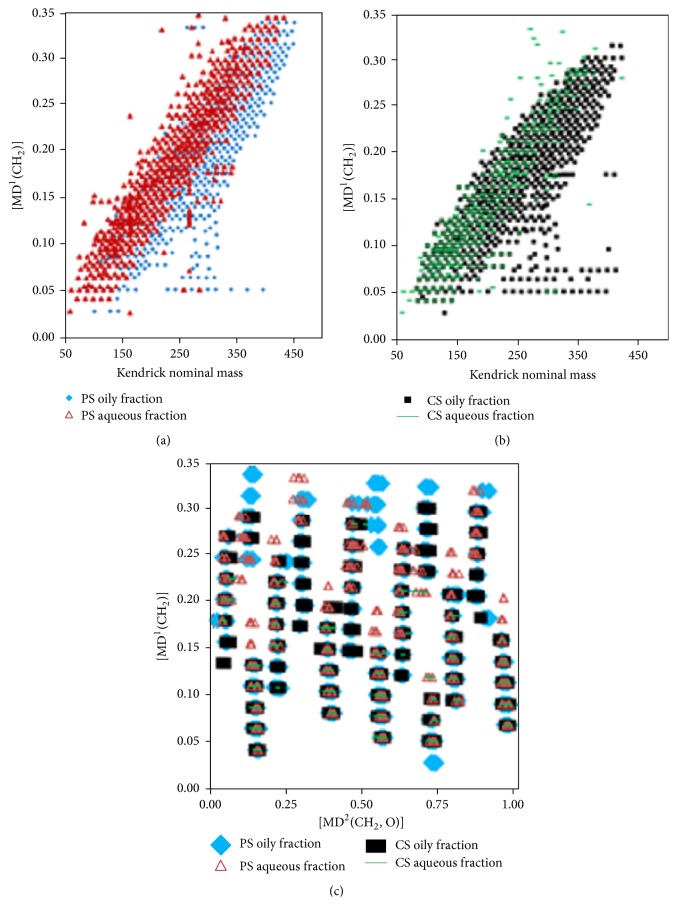
Kendrick mass defect (KMD) analyses for biooil fractions (plotted as a Kendrick mass of CH_2_) [27].

**Figure 4:**
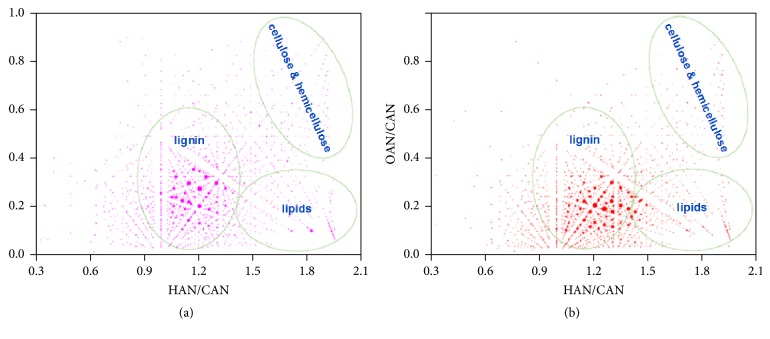
van Krevelen diagrams of O_*n*_ class species by negative-ion ESI FT-ICR MS analysis [28].

**Figure 5:**
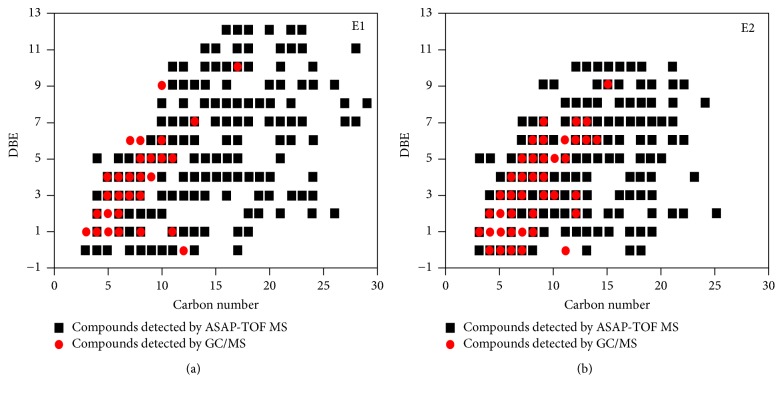
DBE versus carbon number of* n*-hexane and CCl_4_ extractable species in a rice husk biooil using atmospheric solids analysis probe mass spectrometry [29].

FT-ICR MS provides nonspecific identification of molecular species within a wide range of MW (200–1000 Da) with considerable high mass resolution (better than 0.003 Da) and elucidates the detailed composition of complex samples, such as crude oils, biooils, and liquefied products of coals [28, 50, 74, 76, 159, 160]. The species to be determined should be ionized to fragments first by an ionization source without structural changes that could potentially lead to misinterpretation of the acquired data. The produced ions are introduced into a cyclotron (called ICR cell) via a high vacuum pump and then undergo cyclotron motion in a homogeneous magnetic field. After sufficient time, the frequency of the ion with expected high accuracy and precision can be acquired. By using the measured frequency, the* m/z* of specific ion can be calculated. Mass spectrum can be obtained by resolving the frequencies of ions with Fourier transformation. Due to the high mass resolution power of FT-ICR MS, each spectrum may contain thousands of peaks, and the data interpretation for assessment of corresponding species is crucial issue for the elucidation of the componential of complex mixtures. Kendrick mass defect representation (KMD) [27] (see Figure 3), van Krevelen analytical methods [28] (see Figure 4), and DBE versus carbon number plots [29] (see Figure 5) are frequently used to describe the componential features of classes of species contained in samples detected with FT-ICR MS. More than 800 components composed of heteroatom classes from O_2_ to O_14_ with a carbon number of C_6_–C_27_ and a DBE of 1–14 were identified in biooil by using ESI(−)-FT-ICR MS [31]. But only 40 of them were detected with conventional GC-MS. An advantage of the FT-ICR MS analysis is that only dilution or addition of ionization assists reagents for sample pretreatment, which is much simpler compared to conventional chromatographic analysis. Elucidation of components with high MWs is a crucial and difficult challenge for the full understanding of the composition of biooil. Another outstanding advantage of FT-ICR MS is that high MW species and heteroatom-containing compounds can be identified and quantified effectively [47, 150, 161, 162]. FT-ICR MS reveals that this part is mainly composed of poly-oxygenated highly condensed structures, which is important for direction further treatment and evaluation the utilization [52, 163]. However, FT-ICR MS is not competent for the detection of components with low MWs, indicating that conventional chromatographic techniques are necessary for the complete elucidation of biooils [76, 164]. Another factor limiting its application is the high cost of the instrument setup, which impels researchers to search for alternative HRMS techniques.

**Figure 6:**
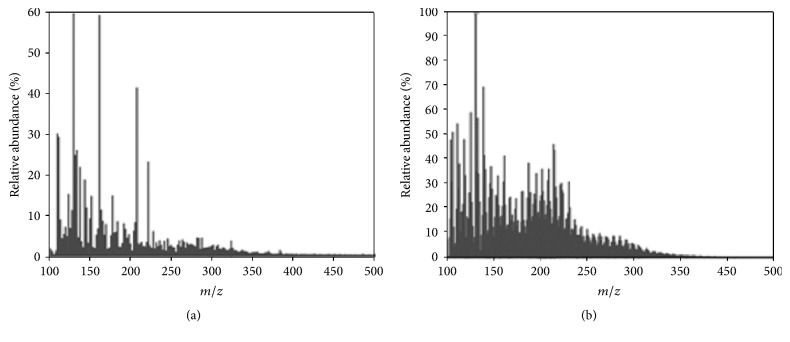
Negative-ion ESI-MS (a) and negative-ion APCI-MS spectra (b) of pyrolysis biooil (Orbitrap Elite;* m*/Δ*m*_50_ = 480,000 at* m*/*z* 400) [30].

In Orbitrap MS, the produced ions from the ionization source are passing throw a series of quadrupoles and deflection lenses before being introduced to analyzer by switching the voltage applied to the deflector lens located on the Orbitrap. In the Orbitrap, ions rotate around the central electrode and oscillate with frequencies of 50–150 kHz for corresponding* m*/*z* of 200–2000 [30, 165]. Alsbou and Helleur [156] carried out a successful componential analysis of species in biooil derived from lignin, cellulose, and forest residue, and levoglucosan, carbohydrates, and lignin derivatives were identified. By using Orbitrap Velos and Orbitrap Elite (in the negative-ion mode of ESI and APCI), significant resolving power was obtained,* that is*, 100000 and 480000 at* m*/*z* 400, and 1900 components were identified in the wood derived biooil [30] (see Figure 6).

TOFMS analysis is fast and sensitive to mass assessment of ions, and the operating principle is very simple. In TOFMS, the produced ions from the ionization source are introduced into the flight tube with the same velocity, and then they are accelerated simultaneously by a pulsed direct-current electric field to a kinetic energy of specific electron volts. The ions fly freely in the high vacuum flight tube to a detector located certain distance away. The* m*/*z* of ions can be calculated based on the proportional relationship to the time required. Masses of ions are measured simultaneously in the process. Compared to FT-ICR MS, Q-TOFMS does not have enough resolution to separate compounds with the same accurate nominal mass. However, Q-TOFMS is a good choice for the effective detection of major components in biooils since it is cheaper and simpler [27, 166, 167].

**Figure 7:**
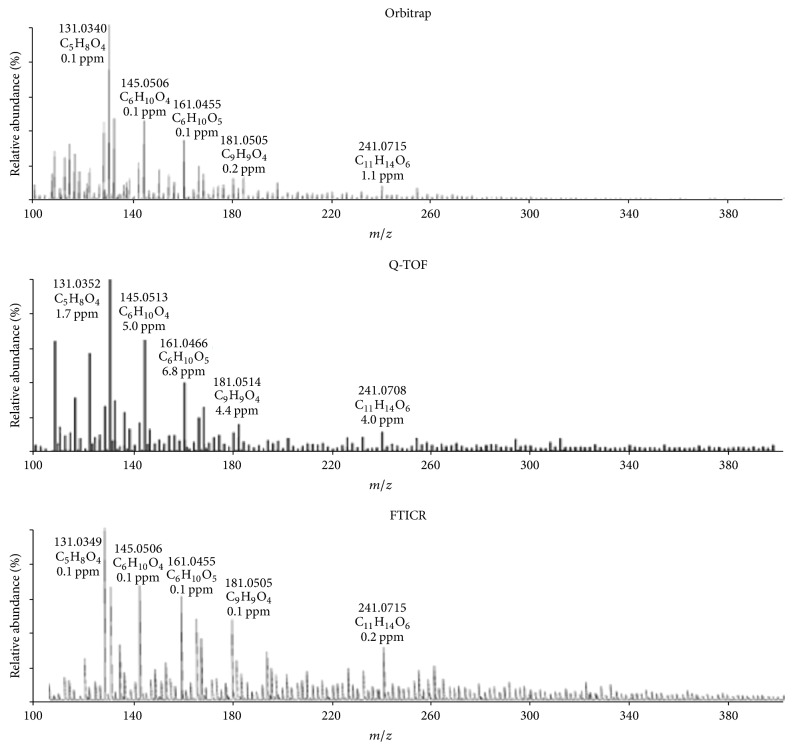
High resolution mass spectra (negative ESI source) of red oak biooils obtained using Orbitrap, Q-TOF, and FT-ICR, respectively [31].

Orbitrap and Q-TOFMS show better discrimination of smaller ions, while FT-ICR MS is more efficient in distinguishing charged fragments with higher* m*/*z*,* that is*, >300 [74, 83]. FT-ICR MS has been proven to be an ideal technique for the deep componential characterization of biooils, since its significant high resolving power for identification thousands of peaks at the level of molecular formula assignment. The choice of MS depending on the requirement of the analysis of complex mixtures, for most of the cases, Orbitrap and Q-TOFMS, can provide satisfactory results, such as the determination of the MW range, the rough assignment of the high MW components, comparison compositional characters between different biooils, and optimization of pyrolytic conditions. FT-ICR MS is a final choice among the HRMS techniques when the detailed and comprehensive analyses of a biooil are required. Orbitrap or TOFMS can be used for the detection of volatile compounds with fewer carbon and oxygen atoms,* that is*, classes from O_0_ to O_8_ with a carbon number of C_3_–C_14_, while with ESI-FT-ICR MS allows the identification of a much broader range of polar, volatile and nonvolatile classes,* that is*, O_2_–O_14_ with carbon numbers of C_6_–C_27_ [74, 76]. More than 8000 and 16000 peaks were identified with ESI(−)-FT-ICR MS in the pine pellet oil and peanut hull biooil, respectively [47]. Typical spectra of HRMS were shown in Figure 7 based on negative ions produced by ESI [31]. Consider the mass resolution and accuracy, the HRMSs follow the order FT-ICR MS > Orbitrap > Q-TOFMS [31, 44, 83].

### 4.5. NMR and Comprehensive NMR (2D NMR)

NMR is a powerful technique for componential characterization of complex mixtures. NMR allows qualitative and quantitative analyses of chemical functionalities and structures in the whole biooil sample without fractionation and assists interpretation of the results of other analytical techniques. Carbon and hydrogen are the main atoms in biooil, so ^1^H and ^13^C NMR were usually used for the determination of their distributions in different structures, such as aliphatic, olefinic, aromatic, methoxy/hydroxy, and carbonyl [168, 169]. ^1^H NMR is the most convenient and extensively spectrometric technique used for the quantification analysis of the major components in biooil [15]. The major components identified in biooil with their typical shifts are as follows: alkanes (0.5–1.6 ppm), aromatics (6.4–7.6 ppm), aldehydes (9.5–10.5 ppm), formic acid (8.10 ppm), acetaldehyde (9.58 and 2.08 ppm), levoglucosan (3.27, 3.84–3.85, 4.31–4.33, and 5.13 ppm), glycolaldehyde (9.55 ppm), hydroxyacetone (4.01 ppm), and acetic acid (1.88 ppm). ^13^C NMR can provide quantitative results of carbon in the various functional groups with complementary information for full characterization of species in fractions of biooil [63]. The ^13^C{^1^H}UDEFT (uniform driven equilibrium Fourier transform) sequence allowing for recording the spectra devoid of heteronuclear NOE (nuclear Overhauser effect) was carried out by Díaz-Urrutia et al. [170] for the characterization of lignin with shorter acquisition times. Different types of carbon atoms in various structures were qualified. After derivatization with 2-chloro-4,4,5,5-tetramethyl-1,3,2-dioxaphospholane, hydroxyl and carboxyl functional groups can be quantified with ^31^P NMR [170]. ^31^P NMR spectra of derived phosphites could also give clear evidence for oxidation of phenolics and lignins to quinines [171–173].

**Figure 8:**
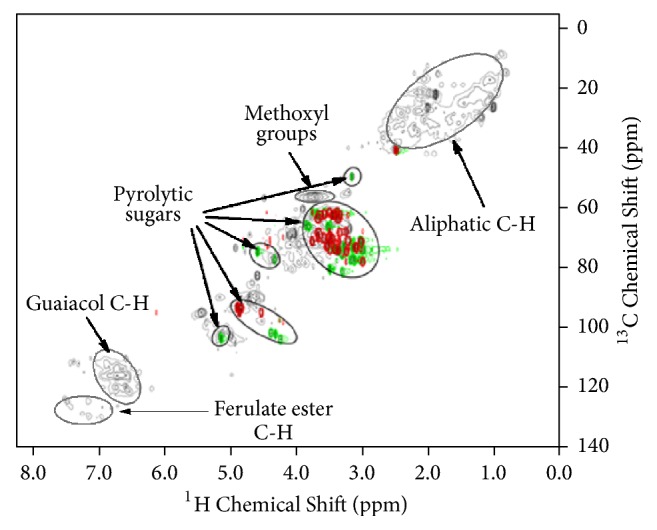
Typical assignments of biooil and various sugar standards in 2D ^1^H-^13^C HSQC spectra. Gray: biooil; red: sugar monomers including glucose, galactose, mannose, xylose, and arabinose; green: anhydrosugars including levoglucosan, cellobiosan, and cellotriosan [32].

2D NMR provides more detailed information on the overview of functional groups and the corresponding quantitative results in biooils without considering MW [174]. Contents of chemical bonds in biooils, such as C-C, C-O, and C-H bonds, could be quantified with 1D NMR and 2D NMR [175]. 2D NMR and solid state 2D NMR have been proven versatile techniques for the structural analysis of lignin and biomass [176, 177]. Heteronuclear single-quantum correlation-nuclear magnetic resonance (HSQC-NMR) was used to characterize the types of C-H bonds and their presence in different moieties of compounds in biooils [32, 57, 75, 164]. 2D ^1^H-^13^C HSQC-NMR was successfully used in the characterization of pyrolytic sugars in fractions of biooil by providing different C-H types in aliphatic, guaiacol, and ferulate structures [32] (see Figure 8). Pyrolysis induces a variety of structural changes to lignin in addition to reduction in MW. In the structural characterization of lignin extracted from the biooil produced by fast pyrolysis of switchgrass* (Panicum virgatum)*, the results of 2D ^1^H-^13^C HSQC-NMR analysis showed the absence of *γ*-methylene hydrogens from *β*-O-4 linkages, implying rearrangements in the propyl linking chains. Ferulate and hydroxyl phenol esters are still present with lower concentrations in pyrolyzed lignin compared to unpyrolyzed switchgrass lignin [56]. The ^1^H-^13^C heteronuclear multiple-bond correlation (HMBC-NMR) spectrum showed that *β*-O-4 linkages, ferulate esters, and guaiacyl ether linkages remained together, indicating significant difference from the pyrolyzed material [56].

## 5. Conclusion and Suggestion

Conventional spectrometric and chromatographic techniques have been applied for the common compositional analysis of biooil allowing detailed determination of its components. FTIR and NMR analyses treat the samples as a whole and provide information on the chemical functional groups and types of chemical bonds. By using conventional HPLC and GC techniques coupled to various detectors, primary identification, and classification of the partial components in biooil with appropriate polarity, MW and boiling point could be obtained. By the combination of pretreatments of sample prior to the chromatographic or spectrometric analyses, the identification of species could be significantly increased. However, more detailed and comprehensive results could not be obtained due to their inherent respective detective limitation, separation capacity, and resolving power of species. The significant development and progress in the comprehensive, hyphenated chromatographic, and spectrometric techniques, such as GC × GC, LC × LC, and 2D NMR, coupled with various MS detectors, have improved the characterization of components in biooil. HRMSs, such as Orbitrap MS, Q-TOFMS, and FT-ICR MS, have been proven effective techniques for the detailed elucidation of components in biooils and complex unknown structures.

With the development of analytical strategies by combining conventional and comprehensive spectrometric and chromatographic techniques, as well as different kinds of HRMSs, significant advancements have been brought for the complete characterization of components in biooils.

## Figures and Tables

**Table 1:** Contents of classes of organic components contained in typical biooils.

Classes of organic components	Contents (%, wt)
Hydrocarbons	1–10
Alcohols	2–5
Furans	1–5
Aldehydes	15–25
Ketones	1–5
Carboxylic acids	5–20
Esters	1–4
Benzene-ring containing species	15–30
Sugars	15–30
Hetroatom-containing species	1–3
Others	1–5

**Table 2:** Typical values for the bulk physicochemical fuel properties of biooil.

Physicochemical fuel properties	Values
Water content	15–30%, wt
Acidity	pH 2–3.5
Density	1.0–1.5 g/mL
Viscosity	10^−3^–10^4^ Pa·s
Heating value	15–25 MJ/kg
Ash content	0.01–0.15%, wt
MW distribution of organic components	30–10000 Da
Mineral contents	10–50 ppm
Solid content	0.2–3.5%, wt
Surface tension	15–30 mM/m (25°C)
Flash point	40–120°C
Elemental contents	C (50–65%), H (5–8%), O (30–40%), N (0.1–0.8%), S (0.01–0.2%), wt

**Table 3:** Selected techniques used in the pretreatment based on properties of components.

Property	Techniques
Solubility	Extraction, precipitation, crystallization, centrifugation
Polarity	Extraction, chromatography
Volatility	Distillation
Density	Sedimentation, centrifugation, flotation
Size and shape	Chromatography, centrifugation, filtration
Electrostatic charge	Chromatography, electrophoresis, flotation
